# Computational Docking as a Tool in Guiding the Drug Design of Rutaecarpine Derivatives as Potential SARS-CoV-2 Inhibitors

**DOI:** 10.3390/molecules29112636

**Published:** 2024-06-03

**Authors:** Shengying Lin, Xiaoyang Wang, Roy Wai-Lun Tang, Ran Duan, Ka Wing Leung, Tina Ting-Xia Dong, Sarah E. Webb, Andrew L. Miller, Karl Wah-Keung Tsim

**Affiliations:** 1 Center for Chinese Medicine, Division of Life Science, The Hong Kong University of Science and Technology, Clear Water Bay, Kowloon, Hong Kong, China; lishlin@ust.hk (S.L.); xwangfr@connect.ust.hk (X.W.); roytwl@ust.hk (R.W.-L.T.); duanran@ust.hk (R.D.); lkwing@ust.hk (K.W.L.); botina@ust.hk (T.T.-X.D.); barnie@ust.hk (S.E.W.); almiller@ust.hk (A.L.M.); 2 State Key Laboratory of Molecular Neuroscience, Division of Life Science, The Hong Kong University of Science and Technology, Clear Water Bay, Kowloon, Hong Kong, China

**Keywords:** viral entry, SARS-CoV-2, rutaecarpine, drug design, structure-activity-relationship study, computational docking

## Abstract

COVID-19 continues to spread around the world. This is mainly because new variants of the SARS-CoV-2 virus emerge due to genomic mutations, evade the immune system and result in the effectiveness of current therapeutics being reduced. We previously established a series of detection platforms, comprising computational docking analysis, S-protein-based ELISA, pseudovirus entry, and 3CL protease activity assays, which allow us to screen a large library of phytochemicals from natural products and to determine their potential in blocking the entry of SARS-CoV-2. In this new screen, rutaecarpine (an alkaloid from *Evodia rutaecarpa*) was identified as exhibiting anti-SARS-CoV-2 activity. Therefore, we conducted multiple rounds of structure-activity-relationship (SAR) studies around this phytochemical and generated several rutaecarpine analogs that were subjected to in vitro evaluations. Among these derivatives, RU-75 and RU-184 displayed remarkable inhibitory activity when tested in the 3CL protease assay, S-protein-based ELISA, and pseudovirus entry assay (for both wild-type and omicron variants), and they attenuated the inflammatory response induced by SARS-CoV-2. Interestingly, RU-75 and RU-184 both appeared to be more potent than rutaecarpine itself, and this suggests that they might be considered as lead candidates for future pharmacological elaboration.

## 1. Introduction

The respiratory infection coronavirus disease 19 (COVID-19), caused by severe acute respiratory syndrome coronavirus 2 (SARS-CoV-2), has had a catastrophic effect on all aspects of our lives on a global scale since the outbreak started in 2019. Various vaccines and oral treatments have been developed to treat COVID-19, and these have been employed worldwide to reduce the rate of infection and hence the course of the disease [1,2,3]. Nevertheless, several variants of concern (VOCs) have emerged, given Greek letter monikers from alpha to omicron, and appear more transmittable and contagious than the initial wild-type virus. Indeed, several lines of evidence indicate that VOCs such as the omicron variant can trigger immune escape, which has resulted in the initial COVID-19 treatments that were developed having a reduced therapeutic effect [4,5]. This suggests that there is still a need for the identification and/or development of novel antagonists that target the variants of SARS-CoV-2 to combat this wide-ranging disease.

The viral entry and replication mechanisms of SARS-CoV-2 have been well documented in previous reports [6,7,8]. In brief, the spike (S)-protein from the virus binds to the angiotensin-converting enzyme 2 (ACE2) receptor on the host cells, and this leads to the virus fusing with the host cell membrane and subsequent invasion. The virus then replicates in the host cells via several key proteins, including 3CL protease, PL protease, and RNA polymerase [6,7]. It is worth noting that, when compared with the original wild-type strain, VOCs contain mutations within the binding proteins, which results in them exhibiting distinct binding affinities, transmissibility, and eventual immune escape from antibodies. For example, approximately 37 residues are mutated within the S-protein of the omicron variant, and 15 of these (including K417N, T478K, and N501Y) occur in the receptor-binding domain (RBD) [8,9,10]. In particular, it was reported that the K417N and E484K mutations were responsible for the immune escape of the omicron variant [9,10]. Together, these proteins form a network to regulate the entry and growth of the virus in the host cells, thereafter resulting in somatic damage to the patient. Thus, the design of an inhibitor that effectively targets one or more protein(s) within this network would be extremely useful as it might lead to the development of potent therapeutics against SARS-CoV-2 and its variants [11].

In drug development, the “hit identification” and “hit-to-lead optimization” phases are crucial in discovering molecules with “drug-likeness”, and they play a fundamental role in the design of new pharmaceuticals. In recent decades, fragment-based approaches have been widely utilized during these two phases [12]. The concept of fragment-based drug discovery (FBDD) was first reported in 1981 by Jencks et al. [13], who proposed that compounds (while normally bound to their target proteins in a relatively weak manner) might also act as small fragments. In this way, they form efficacious interactions and thus allow for further optimization through structure-activity-relationship (SAR) studies, with the aim to grow the chemical scaffolds into highly active lead molecules [14,15]. Interestingly, the structured-based drug design relies on an informative 3D structure between a ligand and a specific target. Molecular docking has become an increasingly popular and efficient tool in predicting and understanding the binding interactions between chemical molecules and biological targets, and this is beneficial to fragment-based drug design and development [16,17].

In our previous reports, we demonstrated that rutaecarpine (a pentacyclic indolopyridoquinazolinone alkaloid from *Evodia rutaecarpa* (Juss.) Benth) inhibited the formation of the S-protein–ACE2 complex and prevented the entry of SARS-CoV-2 into host cells [18]. In addition, rutaecarpine was shown to inhibit the 3CL protease, which suggests that this alkaloid might help to suppress viral replication. Together, our data indicated that rutaecarpine might serve as a potent hit molecule in conducting SAR studies, to identify derivatives with higher efficacy. To guide our SAR investigations, we utilized computational docking stimulation to predict the binding affinities of newly designed rutaecarpine derivatives with the S-protein and 3CL protease. The derivatives were synthesized and evaluated with various in vitro assays, in which they appeared to be relatively potent. Here, we demonstrate that when compared with the parental rutaecarpine molecule, the RU-75 and RU-184 analogs have better efficacy in inhibiting the 3CL protease and preventing viral entry into host cells, as well as attenuating the inflammatory response induced by SARS-CoV-2.

## 2. Results

### 2.1. In Silico-Guided Drug Design of Rutaecarpine Derivatives

**Figure 1:**
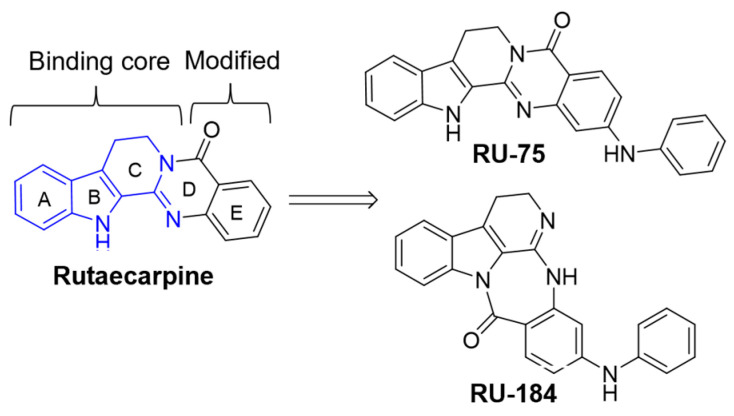
Design of rutaecarpine derivatives. The part of the scaffold shown in blue was identified as the binding core, responsible for the binding interactions, and rings D and E were the parts of the structure modified to yield the RU-75 and RU-184 analogs.

The SAR study commenced with several rounds of in silico screening, where three of the rings of rutaecarpine (labelled A, B, C in Figure 1) were found to act as the binding core. Thus, they were predicted to be responsible for the binding of rutaecarpine to various protein targets, including the 3CL protease, as well as both the wild-type and omicron S-proteins. This allowed us to introduce various substituents in the D and E rings of rutaecarpine to develop active molecules with higher potency, including RU-75 and RU-184 (Figure 1).

**Figure 2:**
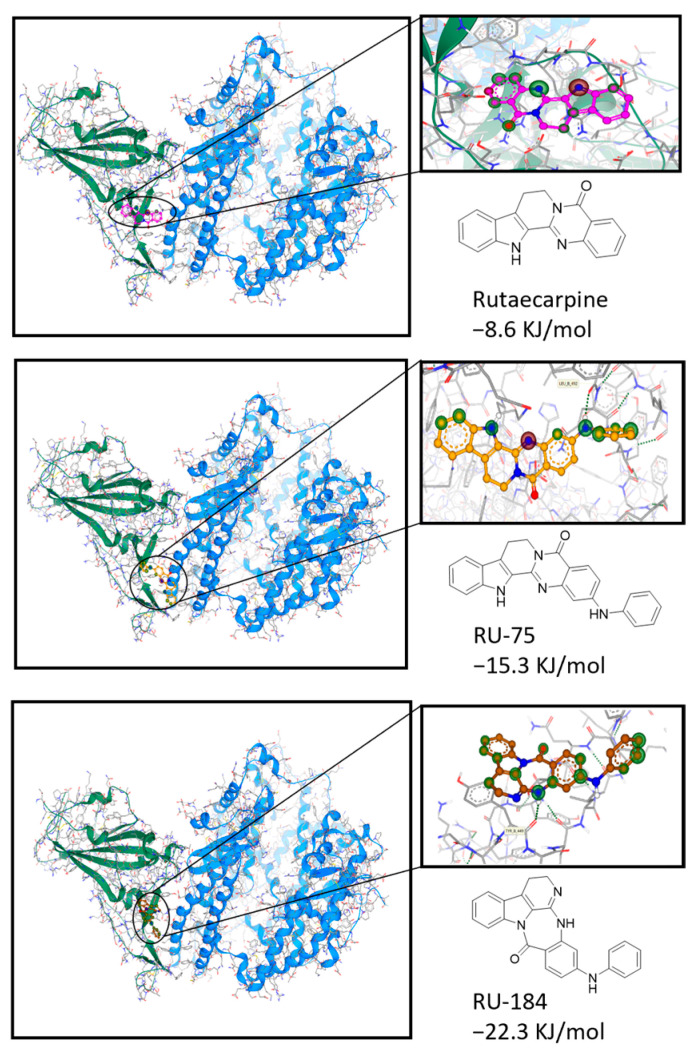
Docking studies to show the interaction of rutaecarpine or its derivatives (RU-75 and RU-184) with the wild-type S-protein. The information about the protein structure was downloaded from the Protein Data Bank (https://www.rcsb.org/, PDB ID: 6LZG, accessed on 28 March 2024), and the chemical structures were obtained from the Pubchem database (https://pubchem.ncbi.nlm.nih.gov/, accessed on 28 March 2024) and Chemdraw (Version 20.0).

As residues 438–506 of the S-protein have been revealed to be the RBD [8,9,10], this enabled us to establish a computational model for our in silico screening. Indeed, when the binding affinities between the newly designed analogs and the RBD were detected, RU-75 and RU-184 showed good interactions at the binding pocket by forming H-bonds with the Leu492 and Tyr449 residues, respectively (Figure 2). Interestingly, the required binding energies of these two molecules were both lower than that of rutaecarpine, with values of approximately −15.3 kJ/mol and −22.3 kJ/mol, respectively. This indicated that RU-75 and RU-184 might display more favorable binding activities at the binding site and might therefore have higher inhibitory activities than rutaecarpine.

**Figure 3:**
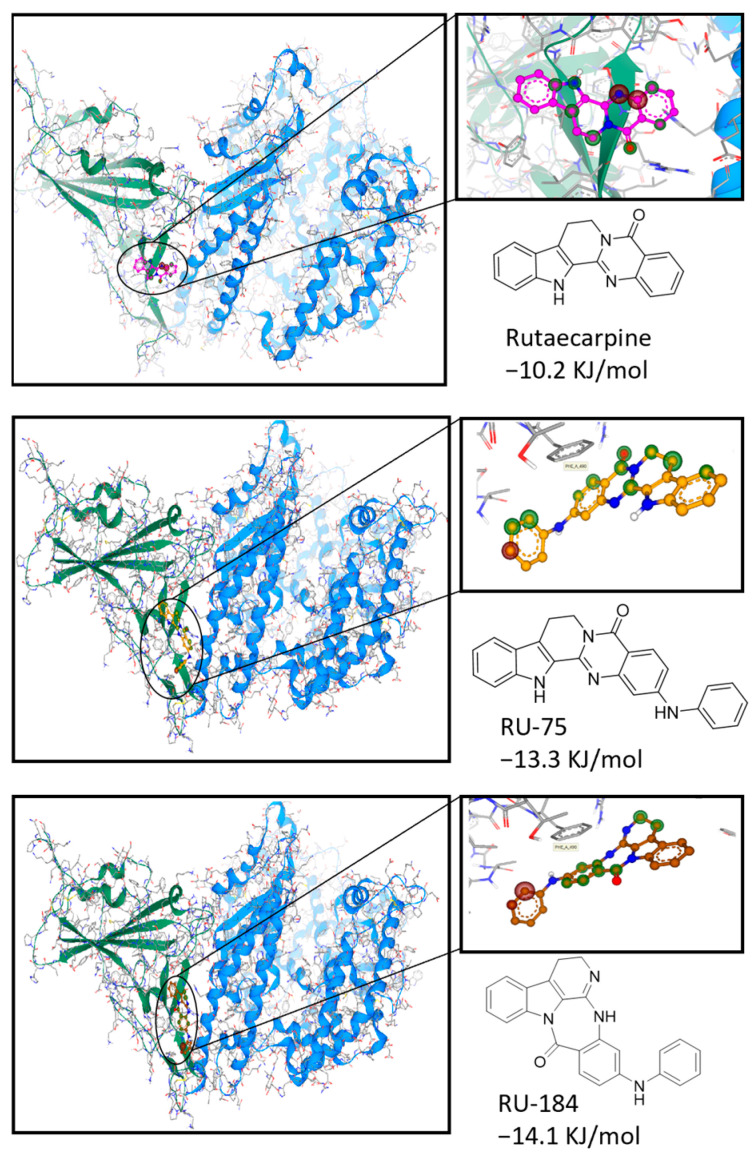
Docking studies to show the interaction of rutaecarpine or its derivatives (RU-75 and RU-184) with the omicron S-protein. The protein structure was downloaded from the Protein Data Bank (https://www.rcsb.org/, PDB ID: 7T9L, accessed on 28 March 2024), and the chemical structures were obtained from the Pubchem database (https://pubchem.ncbi.nlm.nih.gov/, accessed on 28 March 2024) and Chemdraw (Version 20.0).

To evaluate the inhibitory activities of the new analogs against VOCs, the S-protein of the omicron variant was obtained and employed in our docking model. It was observed that RU-75 and RU-184 both bound to the desired pocket of the RBD by forming pi–pi interactions with the Phe490 residue and H-bonds with the Tyr493 residue (Figure 3). As such, both molecules required lower binding energies at the pocket than rutaecarpine. This suggests that both derivatives are likely to inhibit the interaction between the S-protein of the omicron virus and ACE2.

**Figure 4:**
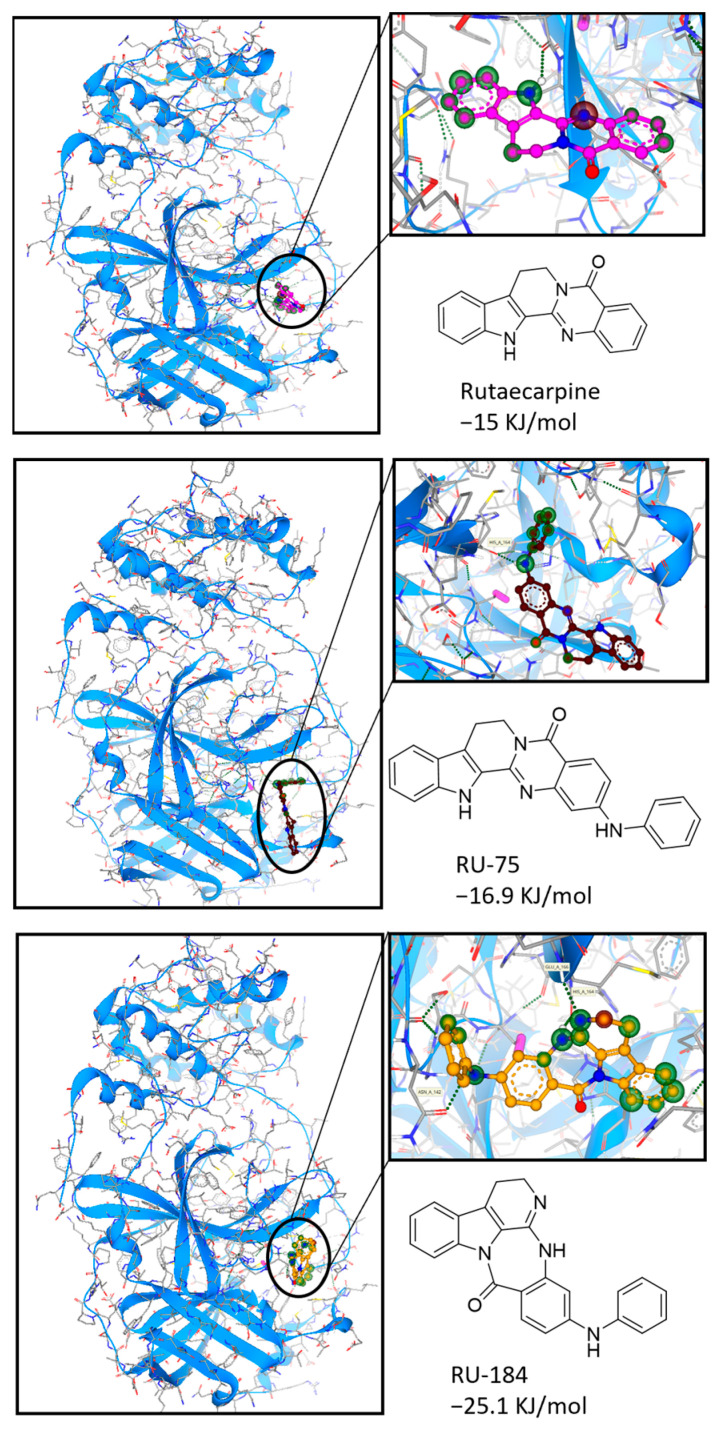
Docking studies to show the interaction of rutaecarpine or its derivatives (RU-75 and RU-184) with the 3CL protease. The protein structure was downloaded from the Protein Data Bank (https://www.rcsb.org/, PDB ID: 6WNP, accessed on 28 March 2024), and the chemical structures were obtained from the Pubchem database (https://pubchem.ncbi.nlm.nih.gov/, accessed on 28 March 2024) and Chemdraw (Version 20.0).

The active site of the 3CL protease has previously been identified, and compounds binding to this site were reported to lead to protein suppression and a reduction in viral replication [19]. In addition, we previously demonstrated, using an in vitro assay, that rutaecarpine could bind to the active site of the 3CL protease, expressing inhibitory effectiveness towards the protein [18]. Here, somewhat intriguingly, it was found that RU-75 formed an extra H-bond with the His164 residue, while RU-184 established H-bonds with the Asn142, His164 and Glu166 residues at the desired pocket. As such, RU-75 and RU-184 were predicted to interact with the active site more favorably and required lower energies than rutaecarpine (Figure 4). For this reason, both derivatives were subjected to organic synthesis prior to further in vitro investigations.

**Figure 5:**
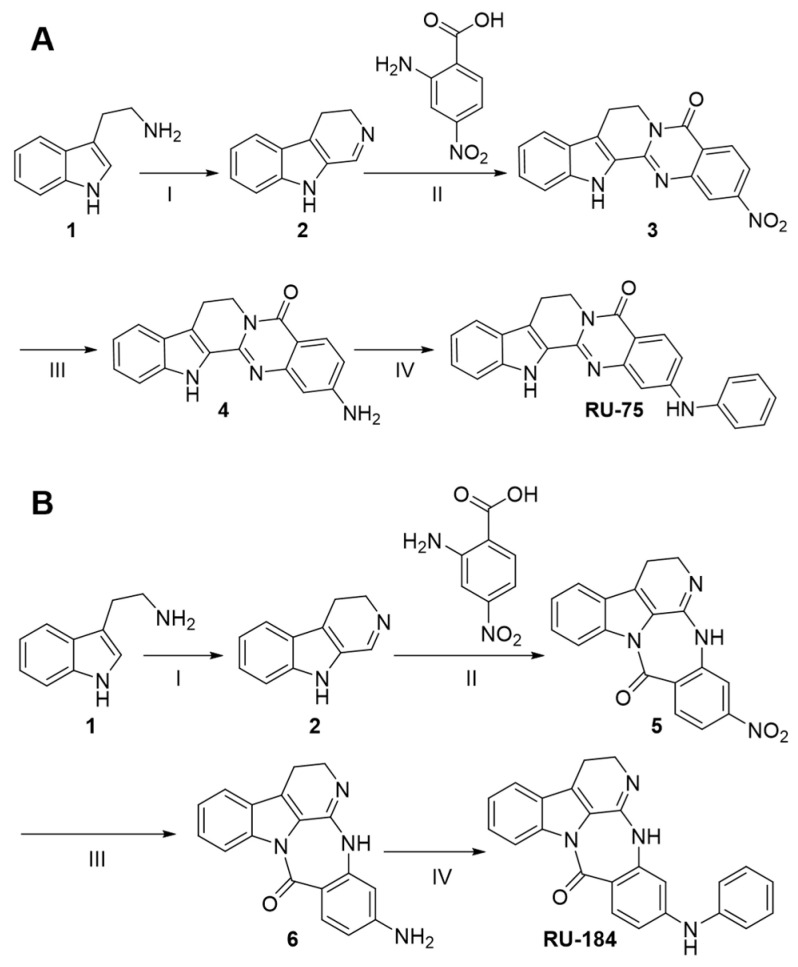
The synthetic routes of RU-75 and RU-184. (**A**) RU-75: reagents and conditions: (I) ethyl formate, MeOH, Et_3_N, 100 °C, 16 h; then POCl_3_, CH_2_Cl_2_, 0 °C-RT, 2 h, 52%; (II) 4-nitroanthranilic acid, EDCI, DMF, 80 °C, 20 h; (III) Fe, NH_4_Cl, EtOH, H_2_O, 80°C, 1 h, 26% (over two steps); (IV) 1-bromobenzene, Pd(OAc)_2_, XPhos, CsCO_3_, dioxane, 100 °C, 20 h, 49%. (**B**) RU-184: reagents and conditions: (I) ethyl formate, MeOH, Et_3_N, 100 °C, 16 h; then POCl_3_, CH_2_Cl_2_, 0 °C-RT, 2 h, 52%; (II) 4-nitroanthranilic acid, EDCI, DMF, 80 °C, 20 h; (III) Fe, NH_4_Cl, EtOH, H_2_O, 80 °C, 1 h, 29% (over two steps); (IV) 1-bromobenzene, Pd(OAc)_2_, XPhos, CsCO_3_, dioxane, 100 °C, 20 h, 57%.

The synthesis pathways of RU-75 and RU-184 are shown in Figure 5. Specifically, compound **2** was obtained from amine **1** via ring closure in the presence of ethyl formate and POCl_3_. During the second ring closure, compounds **3** and **5** were both generated in an one-pot reaction. This is because both nitrogen atoms of intermediate **2** appeared to be equally active in the amide coupling reaction. Thus, good yields of both RU-75 and RU-184 were collected after stepwise nitro reduction and Buchwald–Hartwig amination [20].

### 2.2. In Vitro Evaluations of the Rutaecarpine Analogs

**Figure 6:**
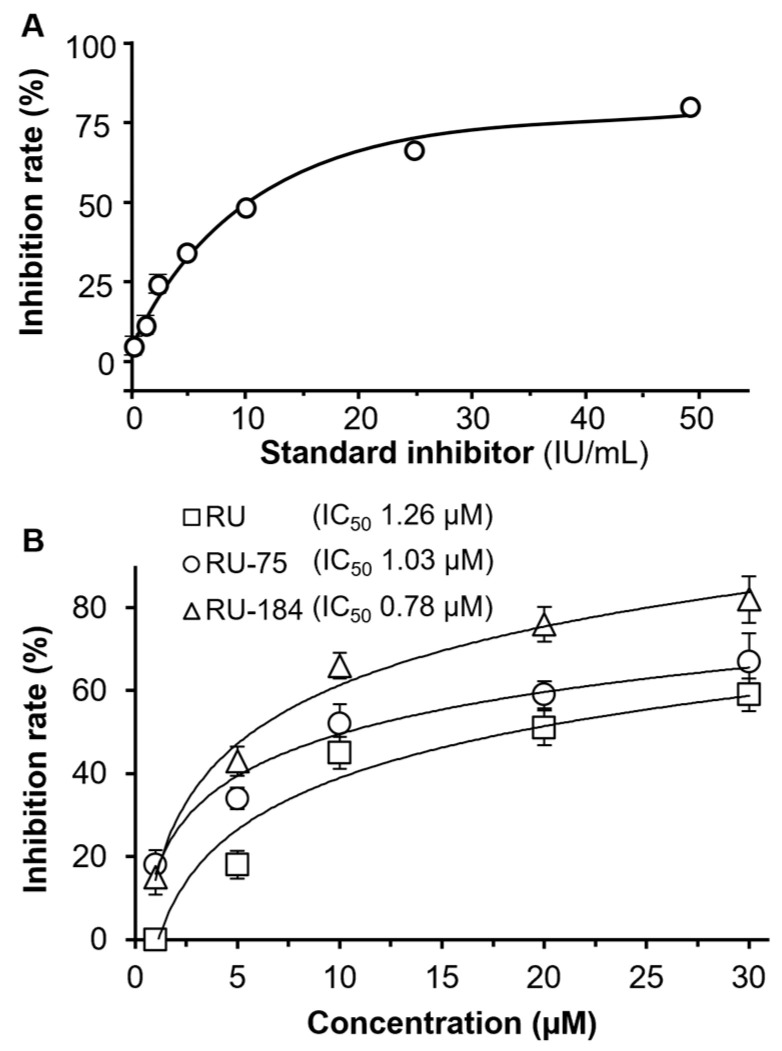
In the S-protein-based ELISA test, RU-75 and RU-184 had a more profound inhibitory effect than RU. (**A**) A standard inhibitor, provided by supplier (calibrated to NIBSC code 20/136), was used as a positive control. (**B**) Rutaecarpine, RU-75 and RU-184 were detected at concentrations of 0.1, 1, 5, 10, 20 µM. The IC_50_ values are shown. The data indicate the mean ± SD percentage of inhibition, compared to a no drug control (*n* = 4).

An S-protein-based ELISA was utilized to determine the binding activities of RU-75 and RU-184 to the S-protein–ACE2 complex. A standard inhibitor, provided by the supplier (calibrated to NIBSC code 20/136), was employed as a positive control (Figure 6A). Our data showed that RU-75 and RU-184 both displayed better inhibitory activities (with IC_50_ values of 1.03 µM and 0.78 µM, respectively) when disrupting the S-protein–ACE2 complex than RU (with an IC_50_ value of 1.26 µM) (Figure 6B). The maximal inhibition, triggered by RU-75 and RU-184, was at least 20% higher than that of RU.

**Figure 7:**
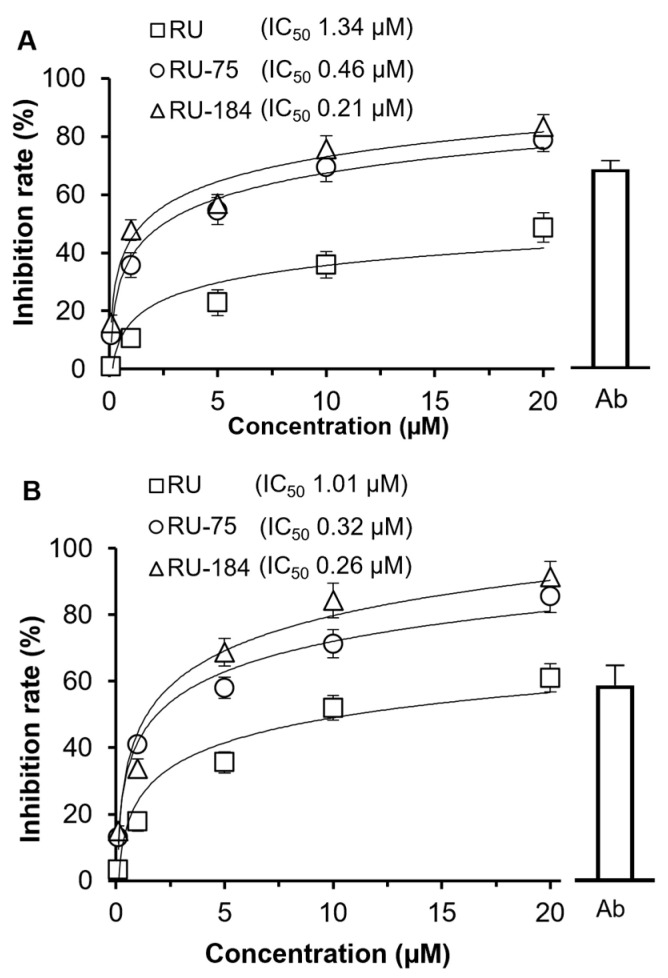
RU-75 and RU-184 block the viral entry of the wild-type (**A**) and omicron (**B**) pseudovirus. Wild-type and omicron antibodies (both used at 1 µg/mL) were used as positive controls for the wild-type and omicron viruses, respectively. RU, RU-75 and RU-184 were tested at concentrations of 0.1, 1, 5, 10, 20 µM. The IC_50_ values are shown. The data indicate the mean ± SD percentage of inhibition, compared to a no drug control (*n* = 5).

Wild-type and omicron pseudovirus cell assays were established to evaluate whether RU-75 and RU-184 might prevent the viral entry of VOCs into host cells. First, an MTT assay was conducted to determine the potential toxicity of the analogs. Our data showed that, similar to rutaecarpine (RU), neither RU-75 nor RU-184 induced significant apoptosis, indicating that they had little impact on the cell viability (Figure S1). In addition, we found that RU-75 and RU-184 both exerted potent efficacy in blocking wild-type viral entry (Figure 7A). The IC_50_ values of RU, RU-75 and RU-184 were 1.34 µM, 0.46 µM and 0.21 µM, respectively. Thus, RU-75 and RU-184 were both more potent inhibitors than their parental compound. Indeed, the maximal inhibition of the analogs was almost double that of RU (Figure 7A). A similar scenario was observed when investigating the effect of RU, RU-75 and RU-184 on the entry of the omicron pseudovirus (Figure 7B). Here, the IC_50_ values of RU, RU-75 and RU-184 were 1.01 µM, 0.32 µM and 0.26 µM, respectively, indicating that once again the analogs were more potent inhibitors than the parent compound.

**Figure 8:**
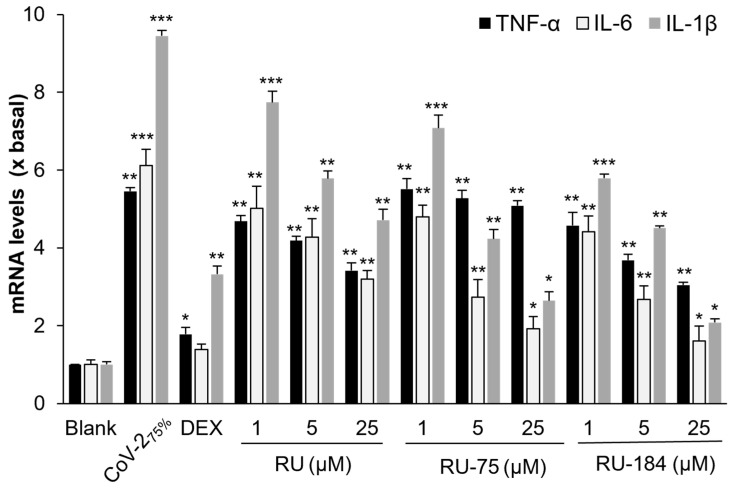
RU-75 and RU-184 attenuate the inflammatory response induced by the wild-type pseudovirus. The mRNA levels of TNF-α, IL-6 and IL-1β were determined by RT-PCR. The virus was used at a concentration of 75% (*v*/*v*), and dexamethasone (DEX) was utilized as the positive control (10 µM). The data show the mean ± SD (*n* = 4) mRNA fold change compared to a blank group (x basal), and the asterisks indicate statistically significant differences such that * *p* < 0.05, ** *p* < 0.01, *** *p* < 0.001 when compared with the blank.

In our previous investigation, we revealed that RU attenuated the inflammatory response induced by SARS-CoV-2 and disrupted the viral entry [16]. Here, we conducted a similar study with RU-75 and RU-184 and found that both of them significantly reduced the production of inflammatory cytokines (TNF-α, IL-6 and IL-1β) in the presence of pseudotyped SARS-CoV-2, when compared with the control group that was treated with the pseudotyped virus alone (Figure 8). In parallel, no significant inflammatory suppression was observed from these molecules when co-treated with lipopolysaccharides (LPS) instead of the pseudotyped SARS-CoV-2 (Figure S2). This suggests that RU-75 and RU-184 might disrupt the interaction between the S-protein and ACE2, leading to the inhibition of viral entry and a subsequent reduction in the inflammatory response.

**Figure 9:**
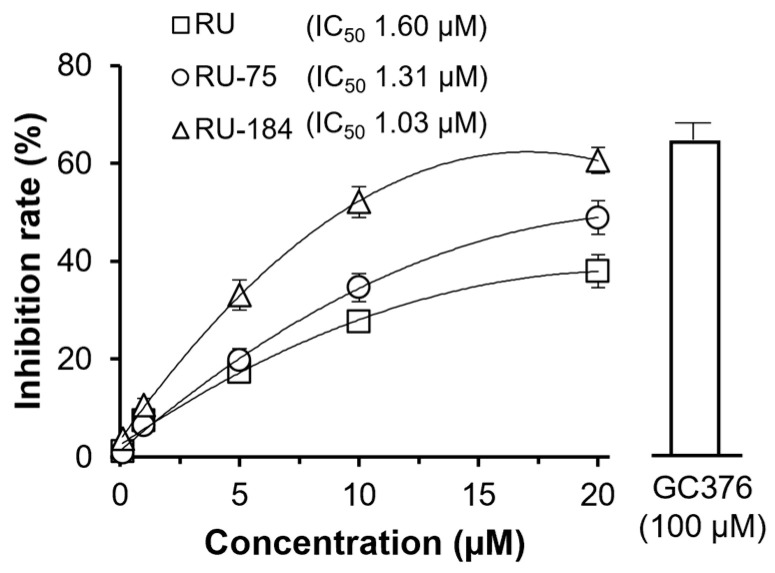
RU-75 and RU-184 suppress 3CL protease activity. GC376 (100 µM) was used as a positive control. RU-75 and RU-184 both inhibited the 3CL protease in a dose-dependent manner. The IC_50_ values are shown. The data indicate the mean ± SD percentage of inhibition, compared to a no drug control (*n* = 4).

In parallel, a 3CL protease assay was carried out to determine the anti-replication effectiveness of both analogs. Indeed, both RU-75 and RU-184 displayed remarkable inhibitory activity towards the protease in a dose-dependent manner, with IC_50_ values of 1.31 µM and 1.03 µM, respectively (Figure 9). Thus, once again, RU-75 and RU-184 both appeared to be more potent than RU (1.60 µM). These findings support our observations on the computational docking modulation. Therefore, we suggest that the two derivatives might bind to the active site of the 3CL protease and, in this way, inhibit the action of this protein, resulting in the potential attenuation of viral replication.

## 3. Discussion

As COVID-19 rapidly spread through communities, a vaccination program was rolled out on a global scale and effectively reduced the rate of infection [21]. In parallel, several oral drugs were developed for clinical application that were shown to possess anti-viral efficacy. For example, the oral medicine molnupiravir was one of the major drugs that was used to treat SARS-CoV-2 patients. It was shown to be effective in attenuating the risk of hospitalization and death and provide relief from symptoms among patients with mild or moderate COVID-19 [22,23]. Nevertheless, the emerging new SARS-CoV-2 VOCs displayed remarkable immune escape abilities and reduced the effectiveness of oral therapies [24,25]. For example, paxlovid was initially identified as being an efficacious anti-COVID-19 treatment, but it was unable to induce fundamental viral clearance and to attenuate the mortality among patients who contracted the omicron variant [26,27]. This indicates that more potent drugs are still required, specifically to tackle these VOCs.

In drug development, there are several crucial phases. These include target identification, target validation, hit identification, and hit-to-lead optimization, as well as pre-clinical and clinical evaluations [28]. Drugs commonly fail in the clinic due to a lack of efficacy and safety issues; therefore, target identification and validation are becoming increasingly vital for drug discovery. Targets of interest include proteins, genes, and RNA, which enable the screening of hit molecules that express good to moderate activity for subsequent optimization. Fragment screening and high-throughput screening are two widely used strategies at this stage to yield hit candidates for subsequent expansion [28,29]. The hit-to-lead phase of drug development typically commences with the determination of structure-activity-relationships to generate a large library of analogs and drive the hit molecules towards compounds with high drug-likeness. Prior to pre-clinical and clinical assessments, lead candidates are subjected to multiple rounds of in vitro assessments and in vivo studies, to identify those that possess superior efficacy and pharmaceutic properties [12,30].

Structural modifications based on rutaecarpine are well documented [31,32,33]. For example, Kim et al. [31] conducted a series of SAR studies and found two rutaecarpine derivatives with improved inhibitory activity against topoisomerase I and II. In addition, Li et al. [32] reported that *N*-aryl/alkyl-substituted rutaecarpine analogs displayed better performance in upregulating ABCA1 activity when compared with the parent compound. In another comprehensive review, Son et al. [33] revealed that rutaecarpine analogs had more potent vasodilator activity than rutaecarpine. This inspired us to carry out intensive SAR investigations on rutaecarpine with the aim to enhance its anti-SARS-CoV-2 activity.

Previously, we established a series of detection assays. One of these was computational docking stimulation, which was found to be a relatively efficient in silico platform allowing us to screen a large library at a low cost. With these detection methods in hand, a number of rutaecarpine derivatives were designed and evaluated, and those displaying relatively good binding affinities to the targeted proteins were selected, synthesized, and evaluated. In this way, RU-75 and RU-184 (derived from rutaecarpine, a hit molecule that expressed good anti-SARS-CoV-2 activity) were generated via structural modification and multiple rounds of SAR studies. Both analogs were found to bind to the targeted proteins (i.e., the wild-type S-protein, omicron S-protein and 3CL protease) in our docking analysis. They also exhibited good potency in the S-protein-based ELISA and 3CL protease assay, as well as the wild-type and omicron pseudovirus entry tests. The two derivatives generally showed lower IC_50_ values and appeared more effective than the parental phytochemical molecule—rutaecarpine. In general, the biological activity obtained from the in vitro investigations was in good agreement with the computational analysis, indicating that the molecular docking methodology was validated, and it efficiently guided the subsequent drug design and drug screening. Taken together, our investigations resulted in two potent lead candidates that might be subjected to follow-up pharmaceutical elaboration in the future, including in-depth pharmacokinetic and pharmacodynamic studies.

## 4. Materials and Methods

### 4.1. Cell Culture

HEK293T and RAW264.7 cells (American Type Culture Collection, Manassas, VA, USA) were cultured and maintained as described previously [34,35], except that the absorbance was measured at 490 nm.

### 4.2. Production of SARS-CoV-2 Pseudotyped Virus

The SARS-CoV-2 pseudotyped virus was produced following previous protocols [34,35]. Plasmids NR-52514 and 179907 were used for the wild-type and omicron SARS-CoV-2 spike glycoproteins, respectively.

### 4.3. Inhibiting SARS-CoV-2 Pseudovirus Entry

ACE2-overexpressing HEK293T cells were seeded into 48-well plates, 400 µL culture medium containing SARS-CoV-2 pseudovirus (100 µL) ± treatment reagents was applied, and the cells were incubated at 37 °C for 24 h. This medium was then replaced with fresh culture medium, and the cultures were allowed to recover for a further 48 h. Immediately prior to the start of the luciferase assay, the cultures were washed with PBS. An anti-SARS-CoV-2 neutralizing antibody (A19215, ABClonal, Woburn, MA, USA) was used as a positive control (1 µg/mL), whereas a solvent blank without the pseudovirus was used as a negative control. The inhibition percentage was determined according to the luciferase activity normalized to the activity of the enzyme without any treatment.

### 4.4. Luciferase Assay

The luciferase assay was conducted as previously described [35]. The percentage inhibition of each sample was calculated as follows:

Inhibition rate = (luciferase activity of the solvent blank − luciferase activity of the sample)/(luciferase activity of the solvent blank − luciferase activity of group without pseudovirus) × 100%.

### 4.5. Inflammation Test

RAW264.7 cells were incubated with a diluted concentration of 75% (*v*/*v*) wild-type SARS-CoV-2 virus or LPS (0.1 µg/mL), and then they were co-treated with various drugs for 24 h. Dexamethasone (DEX) at a concentration of 10 µM was used as a positive control. RNA was then extracted, and the levels of TNF-α, IL-6, and IL-1β mRNA were measured by RT-PCR. The RNA extraction and RT-PCR protocol were previously described [18].

### 4.6. S-Protein Inhibition

Spike (S)-protein inhibition was analyzed with the SARS-CoV-2 spike–ACE2 binding assay kit (ImmunoDiagnostics Ltd., Hong Kong, China), according to the manufacturer’s instructions. The reaction was terminated by the addition of 2 M H_2_SO_4_, and the data were quantified using a microplate reader (FlexStation; Molecular Devices, San Jose, CA). The percentage of inhibition was calculated as follows:

Percentage of inhibition = (PAvg − SAvg)/PAvg × 100%, where PAvg and SAvg are the mean OD values of the positive control and test sample, respectively.

### 4.7. 3CL Protease Assay

Samples were tested for their ability to bind to 3CL protease on a fluorogenic substrate using the SensoLyte SARS-CoV-2 3CL protease assay kit (AnaSpec, San Jose, CA, USA), according to the manufacturer’s instructions. When the 3CL protease became bound to the substrate, the fluorescence was captured using 360 nm excitation and 460 nm detection. The percentage of inhibition was calculated as follows:

Percentage of inhibition = (PAvg, b − SAvg, b)/PAvg, b × 100%, where PAvg, b and SAvg, b are the mean fluorescence of the positive control and test samples, respectively, subtracted from the mean fluorescence of a blank.

### 4.8. Computational Docking Analysis

The chemical structure of each phytochemical was downloaded from Pubchem, and the protein structures were downloaded from the Protein Data Bank (https://www.rcsb.org/ (accessed on 28 March 2024). Virtual screening was performed with SEESAR (Version 12.0, https://www.biosolveit.de/ (accessed on 28 March 2024). The docking analysis was performed as reported previously [16,36].

### 4.9. Chemistry

Synthesis of RU-75: To a solution of 1-bromobenzene (1.2 eq.), Pd(OAc)_2_ (0.05 eq.), XPhos (0.1 eq.), CsCO_3_ (1.5 eq.) in anhydrous dioxane (5 mL/mmol), amine **4** (1 eq.) was added. The reaction mixture was degassed for 5 min, and then it was heated at 100 °C for 20 h. The mixture was then cooled to room temperature, filtered through celite, and dried under reduced pressure to yield the crude compound, which was purified by normal-phase medium pressure liquid chromatography (MPLC) (EtOAc–petroleum ether) to yield RU-75. ^1^H NMR (300 MHz, DMSO-*d*_6_) δ 3.15 (t, *J* = 6.0 Hz, 2H), 4.40 (t, *J* = 6.0 Hz, 2H), 7.03–7.09 (m, 4H), 7.19–7.28 (m, 4H), 7.37–7.41 (m, 2H), 7.64 (dd, *J* = 1.2 and 4.5 Hz, 1H), 7.96 (d, *J* = 4.5 Hz, 1H), 8.93 (br s, 1H), 11.78 (br s, 1H). ^1^C NMR (101 MHz, DMSO-*d*_6_) δ 19.5, 40.9, 107.5, 112.5, 112.9, 115.9, 118.0, 120.1, 120.3, 120.5 (2C), 122.8, 125.0, 125.4, 127.8 (2C), 128.5, 139.8, 139.0, 141.6, 146.0, 149.7, 150.2, 160.4. HRMS detection for [M+H]^+^: found 379.3105.

Synthesis of RU-184: To a solution of 1-bromobenzene (1.2 eq.), Pd(OAc)_2_ (0.05 eq.), XPhos (0.1 eq.), CsCO_3_ (1.5 eq.) in anhydrous dioxane (5 mL/mmol), amine **6** (1 eq.) was added. The reaction mixture was degassed for 5 min and then heated at 100 °C for 20 h. The mixture was then cooled to room temperature, filtered through celite, and dried under reduced pressure to yield the crude compound, which was purified by MPLC (EtOAc–petroleum ether) to yield RU-184. ^1^H NMR (300 MHz, DMSO-*d*_6_) δ 3.13 (t, *J* = 6.0 Hz, 2H), 4.38 (t, *J* = 6.0 Hz, 2H), 7.05–7.11 (m, 3H), 7.21–7.28 (m, 4H), 7.37–7.42 (m, 3H), 7.62 (dd, *J* = 1.2 and 4.5 Hz, 1H), 7.96 (d, *J* = 4.5 Hz, 1H), 9.02 (br s, 1H), 11.79 (br s, 1H). ^1^C NMR (101 MHz, DMSO-*d*_6_) δ 19.5, 40.8, 107.6, 112.5, 113.0, 115.9, 118.0, 120.1, 120.5 (2C), 121.3, 122.8, 125.0, 125.4, 127.8, 128.5, 129.8 (2C), 139.0, 141.6, 146.0, 149.7, 150.2, 160.5. HRMS detection for [M+H]^+^: found 379.3048.

Synthesis of intermediate **2**: (i) Tryptamine (1.0 eq.), ethyl formate (14 eq.), anhydrous methanol (3 mL/mmol) and triethylamine (1.0 eq.) were added to a 120 mL round-bottomed flask and heated to 100 °C for 16 h, after which it was cooled to room temperature and concentrated under reduced pressure. The residue was dissolved in CH_2_Cl_2_ (5 mL/mmol) and washed with aqueous HCl (1.0 M, 5 mL/mmol), saturated aqueous NaHCO_3_ (5 mL/mmol) and brine (5 mL/mmol). The combined organic layers were dried with sodium sulfate and filtered and concentrated under reduced pressure to yield a crude intermediate, *N*-[2-(1H-indol-3-yl)ethyl]formamide. (ii) POCl_3_ (0.25 mL/mmol) was added dropwise at ~0–5 °C to a solution of intermediate *N*-[2-(1H-indol-3-yl)ethyl]formamide (1 eq.) in CH_2_Cl_2_ (1 mL/mmol). The reaction mixture was stirred at room temperature for another 2 h and concentrated under reduced pressure. The reaction residue was dissolved in EtOAc (10 mL/mmol) and extracted with 10% AcOH in water (10 mL/mmol) three times. The combined AcOH layers were basified with concentrated aqueous ammonia to reach pH 9. The precipitated solid was further washed with CH_2_Cl_2_ (3 × 10 mL/mmol) to yield intermediate **2** without further purification. ^1^H NMR (300 MHz, DMSO-*d*_6_) δ 3.15 (t, *J* = 6.0 Hz, 2H), 3.90 (t, *J* = 6.0 Hz, 2H), 7.13–7.17 (m, 1H), 7.37–7.39 (m, 1H), 7.54–7.56 (m, 1H), 7.71 (d, *J* = 7.8 Hz, 1H), 8.93 (s, 1H), 12.32 (br s, 1H). ^1^C NMR (101 MHz, DMSO-*d*_6_) δ 19.1, 43.2, 113.8, 121.5 (2C), 121.9, 122.4, 124.2, 126.8, 128.2, 140.8. HRMS detection for [M+H]^+^: found 171.2478.

Synthesis of compound **3**: EDCI (1.4 eq.) was added to a solution of compound **2** (1 eq.) and 4-nitroanthranilic acid (1 eq.) in DMF (2 mL/mmol) and heated to 80 °C. The mixture was stirred at this temperature until no starting material was detected by thin layer chromatography (TLC). The reaction was quenched with water (5 mL/mmol) and the resulting suspension was filtered. The precipitated solid was successively washed with water (50 mL/mmol) and ethanol (15 mL/mmol) and then it was dried under reduced pressure. The resulting crude product was recrystallized in CH_2_Cl_2_ and purified by MPLC (EtOAc–petroleum ether) to yield two crude fractions that contained **3** and **5**, respectively. Both crude compounds were subjected to nitro reduction to yield amines **4** and **6**, respectively. Compound **3**: ^1^H NMR (300 MHz, DMSO-*d*_6_) δ 3.10 (t, *J* = 6.0 Hz, 2H), 4.40 (t, *J* = 6.0 Hz, 2H), 7.01 (dd, *J* = 2.1 and 7.2 Hz, 1H), 7.15–7.29 (m, 2H), 7.40–7.49 (m, 2H), 7.51–7.57 (m, 2H), 9.89 (br s, 1H). ^1^C NMR (101 MHz, DMSO-*d*_6_) δ 20.1, 45.8, 112.7, 117.0, 117.9, 118.5, 119.4, 120.6, 122.0, 125.1, 125.9, 126.5, 129.4, 136.7, 145.5, 148.6, 151.7, 160.2. Compound **5**: ^1^H NMR (300 MHz, DMSO-*d*_6_) δ 2.98 (t, *J* = 6.0 Hz, 2H), 4.09 (t, *J* = 6.0 Hz, 2H), 7.05–7.14 (m, 2H), 7.56–7.62 (m, 2H), 7.68–7.71 (dd, *J* = 1.5 and 7.2 Hz, 1H), 7.82–7.89 (m, 2H), 10.12 (br s, 1H). ^1^C NMR (101 MHz, DMSO-*d*_6_) δ 20.9, 45.1, 111.4, 113.6, 115.9, 117.4, 119.2, 120.7, 122.8, 125.1, 126.4, 130.5, 136.0, 143.2, 146.1, 149.5, 150.4, 161.3.

Synthesis of compound **4**: Iron powder (5 eq.) was added to a solution of compound **3** (1 eq.) in anhydrous EtOH (6 mL/mmol), after which NH_4_Cl (5 eq.) in H_2_O (5 mL/mmol) was added. The mixture was stirred at 80 °C for 1 h, cooled to room temperature, filtered through celite, and washed with MeOH (10 mL/mmol) three times. The filtrate was concentrated under reduced pressure to yield the crude compound, which was purified by reverse-phase MPLC (MeCN: H_2_O). ^1^H NMR (300 MHz, DMSO-*d*_6_) δ 3.11 (t, *J* = 6.0 Hz, 2H), 4.35 (t, *J* = 6.0 Hz, 2H), 6.10 (br s, 2H), 6.69–6.74 (m, 2H), 7.09 (dd, *J* = 1.5 and 6.6 Hz, 1H), 7.23 (dd, *J* = 1.8 and 6.0 Hz, 1H), 7.47 (d, *J* = 1.8 Hz, 1H), 7.63 (d, *J* = 2.4 Hz, 1H), 7.81 (d, *J* = 2.4 and 6.0 Hz, 1H), 11.74 (br s 1H). ^1^C NMR (101 MHz, DMSO-*d*_6_) δ 19.6, 40.6, 106.9, 110.1, 112.9, 114.8, 117.6, 120.3 (2C), 124.9, 125.4 (2C), 127.9, 128.3, 138.9, 145.6, 154.8, 160.6. HRMS detection for [M+H]^+^: found 303.1057.

Synthesis of **6**: Iron powder (5 eq.) was added to a solution of compound **5** (1 eq.) in anhydrous EtOH (6 mL/mmol), after which NH_4_Cl (5 eq.) in H_2_O (5 mL/mmol) was added. The mixture was stirred at 80 °C for 1 h, cooled to room temperature, filtered through celite, and washed with MeOH (10 mL/mmol) three times. The filtrate was concentrated under reduced pressure to yield the crude compound, which was purified by reverse-phase MPLC (MeCN: H_2_O). ^1^H NMR (300 MHz, DMSO-*d*_6_) δ 3.13 (t, *J* = 6.0 Hz, 2H), 4.37 (t, *J* = 6.0 Hz, 2H), 6.10 (br s, 2H), 6.71–6.74 (m, 2H), 7.07 (dd, *J* = 1.5 and 6.6 Hz, 1H), 7.25 (dd, *J* = 1.8 and 6.0 Hz, 1H), 7.49 (d, *J* = 1.8 Hz, 1H), 7.65 (d, *J* = 2.4 Hz, 1H), 7.83 (d, *J* = 2.4 and 6.0 Hz, 1H), 11.72 (br s 1H). ^1^C NMR (101 MHz, DMSO-*d*_6_) δ 19.5, 39.3, 107.0, 110.1, 113.0, 114.8, 117.7, 120.2, 120.4, 124.8, 125.4 (2C), 128.0, 128.4, 138.9, 145.6, 154.9, 160.6. HRMS detection for [M+H]^+^: found 303.1687.

## 5. Conclusions

Although the global vaccine program has been widely deployed and several oral treatments have been developed, COVID-19 continues to be transmitted rapidly throughout communities due to the emerging variants of SARS-CoV-2, such as the omicron variant. Rutaecarpine was previously found to exert good anti-SARS-CoV-2 effectiveness and served as a hit molecule for structural modifications. Here, we employed computational docking to guide the drug design of rutaecarpine derivatives; these derivatives were subjected to drug screening for the following in vitro investigations. After multiple rounds of SAR studies, we identified that RU-75 and RU-184 exhibited better anti-viral efficacy than the parental molecule by disrupting the S-protein–ACE2 complex (including the wild-type and omicron variant of SARS-CoV-2), blocking viral entry and inhibiting the 3CL protease. Therefore, these two analogs might prove to be beneficial for subsequent pharmaceutical development as prospective new potent anti-COVID-19 treatments.

## Data Availability

The data presented in this study are available upon request from the corresponding author.

## Supplementary Materials

The following supporting information can be downloaded at: https://www.mdpi.com/article/10.3390/molecules29112636/s1, Figure S1: Testing the cell toxicity of rutaecarpine, RU-75, RU-184 with the MTT cell viability test; Figure S2: RU-75 and RU-184 do not affect the inflammatory response induced by LPS; Figure S3: ^1^H NMR, ^13^C NMR spectra of RU-75; Figure S4: ^1^H NMR, ^13^C NMR and NOE spectra of RU-184. Figure S5: ^1^H NMR, ^13^C NMR spectra of intermediate **2**; Figure S6: ^1^H NMR, ^13^C NMR spectra of intermediate **4**; Figure S7: ^1^H NMR, ^13^C NMR spectra of intermediate **6**.
