# Supplementary material for: Computational Docking as a Tool in Guiding the Drug Design of Rutaecarpine Derivatives as Potential SARS-CoV-2 Inhibitors
Source: Molecules. 2024 Jun 3;29(11):2636. doi: 10.3390/molecules29112636 (PMC11173897; doi:10.3390/molecules29112636)
Supplement: Supplementary file 1 [file molecules-29-02636-s001.zip › molecules-3038897-supplementary.pdf]

Computational docking as a tool in guiding drug design of rutaecarpine derivatives as potential SARS-CoV-2 inhibitors

Shengying Lin[a,b], Xiaoyang Wang[a,b], Roy Wai-Lun Tang[a,b], Ran Duan[a,b], Ka Wing Leung[a,b], Tina Ting-Xia Dong[a,b], Sarah E. Webb[a,b], Andrew L. Miller[a,b], and Karl Wah-Keung Tsim[a,b]

[a]Center for Chinese Medicine and [b]State Key Laboratory of Molecular Neuroscience, Division of Life Science, The Hong Kong University of Science and Technology, Clear Water Bay, Kowloon, Hong Kong, China.

Correspondence

Prof. Karl Wah-Keung Tsim, Center for Chinese Medicine, Division of Life Science, The Hong Kong University of Science and Technology, Clear Water Bay, Hong Kong, China
Phone: +852 2358 7332
Fax: +852 2358 1552
Email: botsim@ust.hk

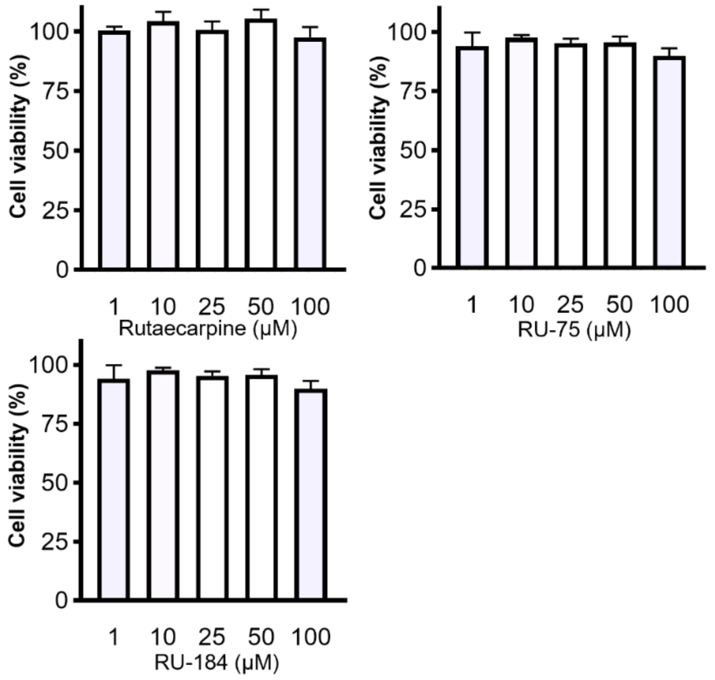

Figure S1. Testing the cell toxicity of rutaecarpine, RU-75, RU-184 with the MTT cell viability test. MTT solution was applied to cells in a final concentration of 0.5 mg/mL (20 μL/well). The optical density of each well was determined at 492 nm. The data indicate the mean ± SD cell viability percentage, compared to a no drug control, (n = 4).

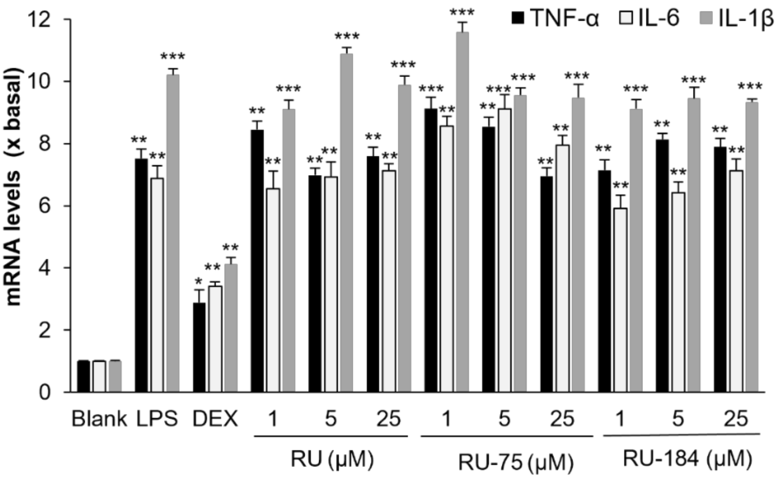

Figure S2. RU-75 and RU-184 do not affect the inflammatory response induced by LPS. The mRNA levels of TNF-α, IL-6, and IL-1β were determined by RT-PCR. LPS was used at a concentration of 0.1 μg/mL, and dexamethasone (DEX) was utilized as the positive control (10 μM). The data indicate the mean ± SD fold change compared to a blank group (x basal), and the asterisks indicate statistically significant differences such that * $p < 0.05$, ** $p < 0.01$, *** $p < 0.001$ when compared with the blank (n = 4).

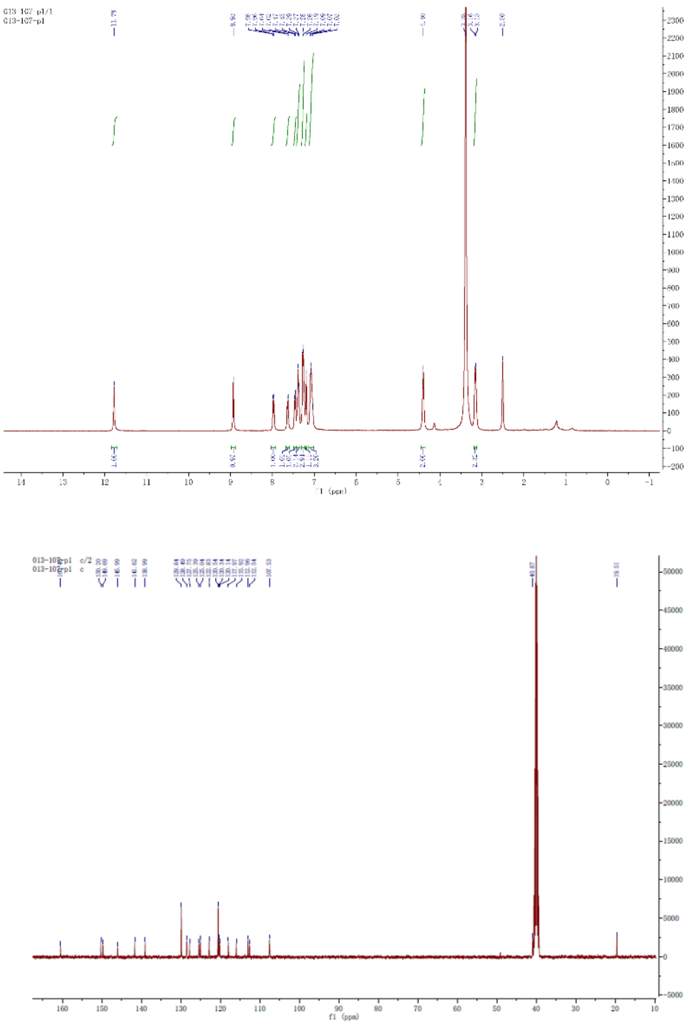

Figure S3. ^{1}H NMR, ^{13}C NMR spectra of RU-75.

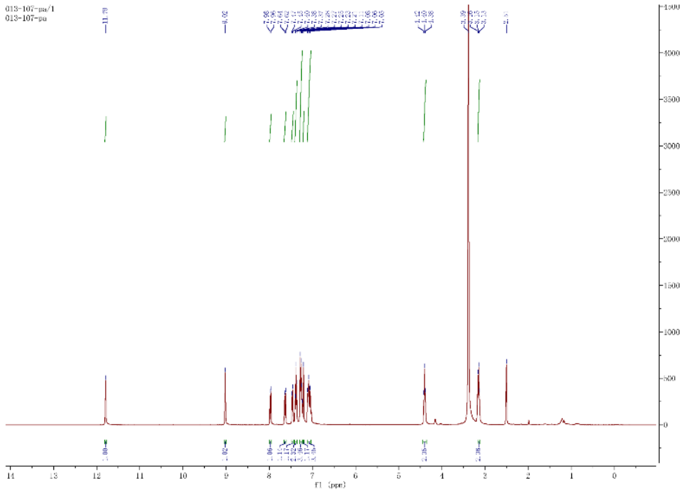

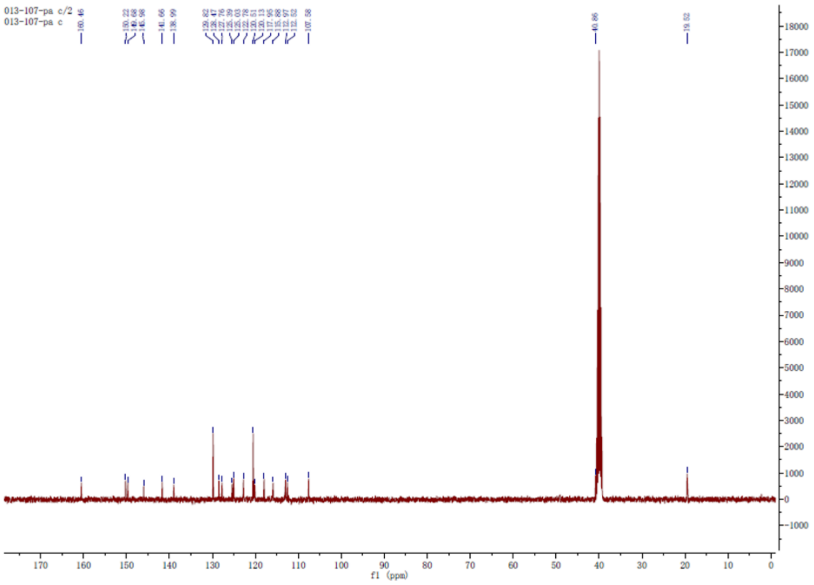

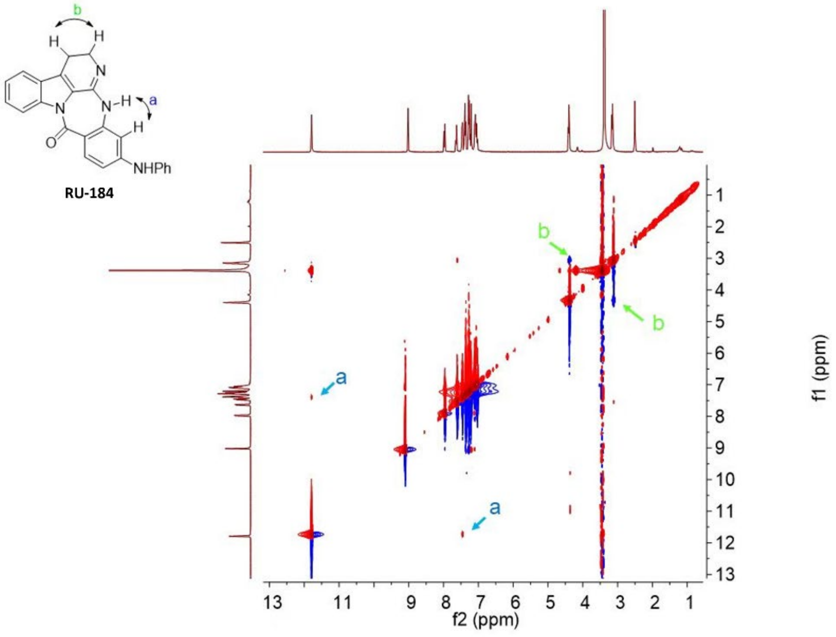

Figure S4. ^{1}H NMR, ^{13}C NMR and NOE spectra of RU-184.

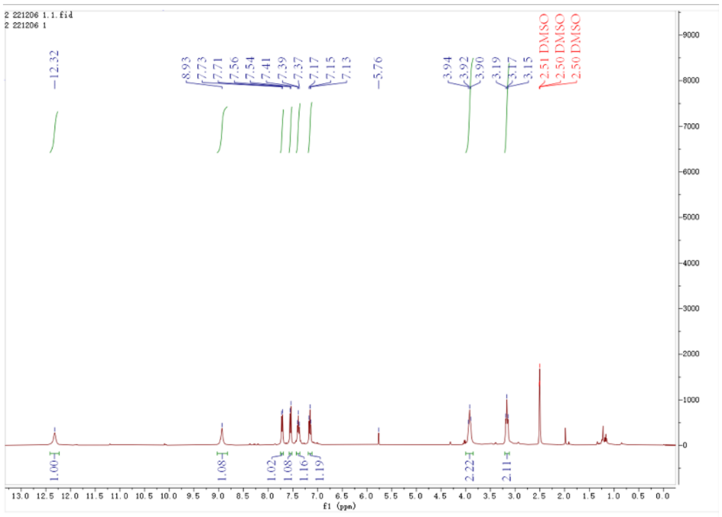

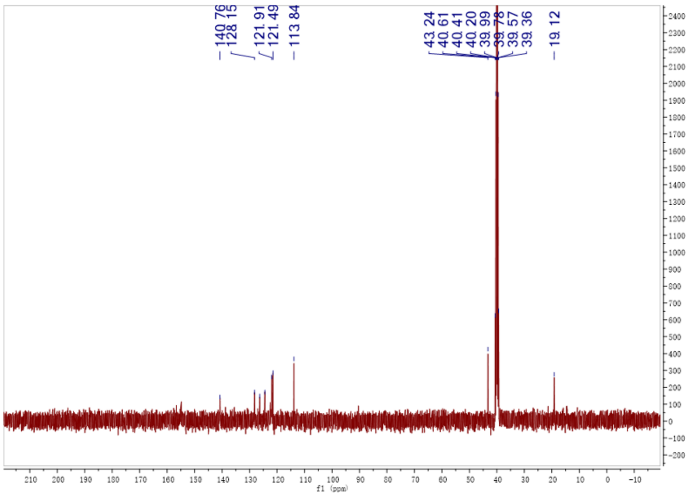

Figure S5. ¹H NMR, ¹³C NMR spectra of intermediate **2**.

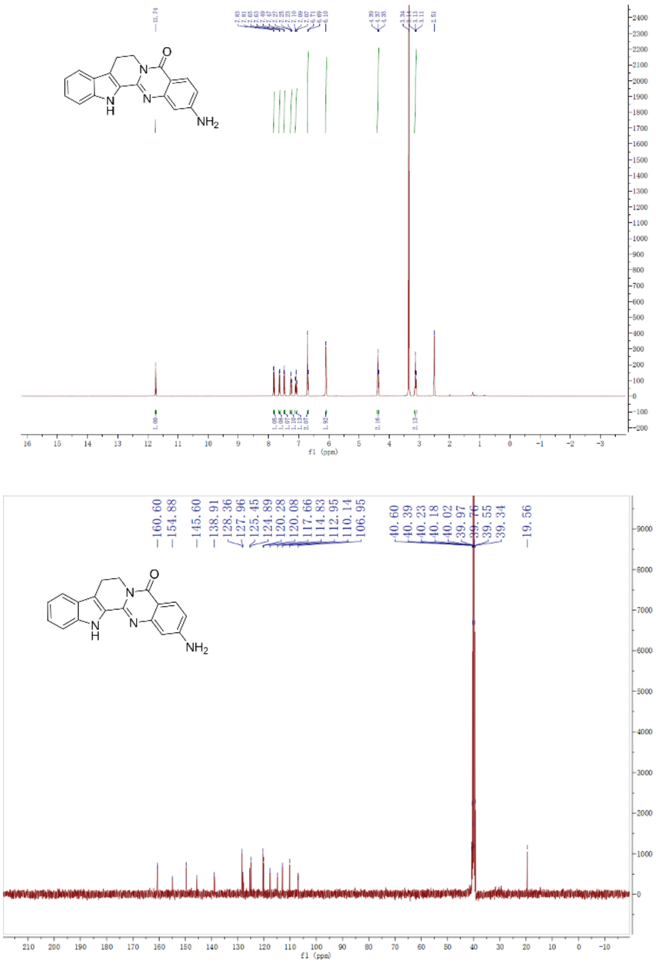

Figure S6. ¹H NMR, ¹³C NMR spectra of intermediate **4**.

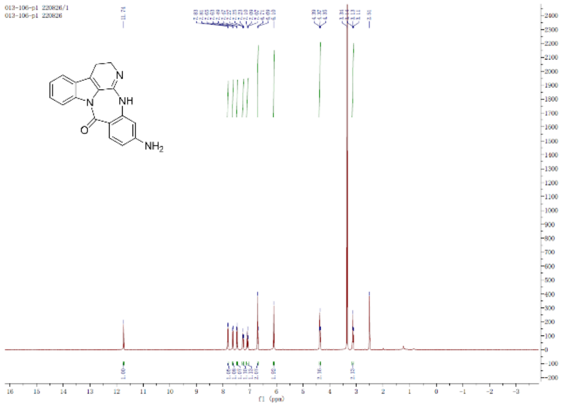

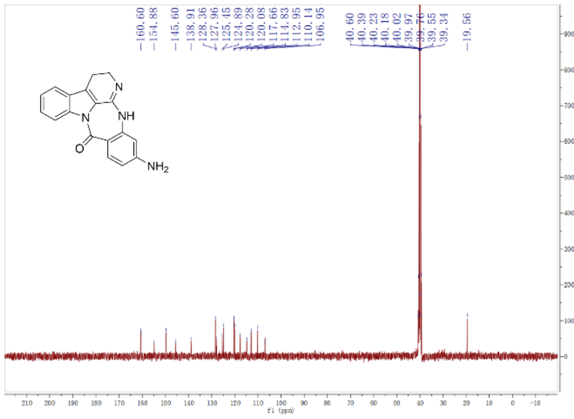

Figure S7. ^{1}H NMR, ^{13}C NMR spectra of intermediate **6**.
